# Multiparametric MRI radiomics integrating Arterial Spin Labeling, Quantitative Susceptibility Mapping, and Amide Proton Transfer imaging for differentiating glioma recurrence from post-treatment response

**DOI:** 10.3389/fonc.2026.1956583

**Published:** 2026-09-17

**Authors:** Qi Wang, Zhenguo Yuan, Yanzhao Diao, Youjiao Si, Shuai Dong, Xiang Lv, Hexin Liang

**Affiliations:** 1 Department of Nuclear Medicine, Jeonbuk National University Medical School and Hospital, Jeonju, Republic of Korea; 2 Department of Radiology, Shandong Provincial Hospital Affiliated to Shandong First Medical University, Jinan, China; 3 Department of Medical Imaging, Guangdong Second Provincial General Hospital, Guangzhou, China; 4 Department of Radiology, Shandong Cancer Hospital and Institute, Shandong First Medical University and Shandong Academy of Medical Sciences, Jinan, Shandong, China

**Keywords:** Amide Proton Transfer Weighted imaging, Arterial Spin Labeling, glioma, Quantitative Susceptibility Mapping, radiomics

## Abstract

**Background:**

Using multimodal MRI, we developed a predictive model for identifying glioma recurrence and treatment response.

**Methods:**

Clinical data were retrospectively collected on 274 patients with gliomas following surgery. All patients underwent the MRI scan, including Contrast-enhanced T1 Weighted Imaging (CE-T1WI), Arterial Spin Labeling (ASL), Amide Proton Transfer Weighted (APTW) imaging, Quantitative Susceptibility Mapping (QSM), and Diffusion-Weighted Imaging (DWI). The radiomics features extracted from these images were used to construct five single sequence models, one combination model, and a Nomogram, aiming to compare the performance of various models in recognizing glioma recurrence and treatment response.

**Results:**

As a result of combining age, maximum tumor diameter, and IDH1 genotype, a clinical model was constructed, and the area under the curve (AUC) value in the training cohort was 0.748, validation cohort was 0.721 and temporal test cohort was 0.706. The AUC for the five single-sequence models ranged from 0.781-0.899 in the training cohort, from 0.705-0.789 in the validation cohort and from 0.708-0.828 in temporal test cohort. The AUC of the combined model was 0.927, 0.934 and 0.968 in the training cohort, validation cohort and temporal test cohort, respectively. The AUC of the nomogram was 0.983 in the training cohort, 0.958 in the validation cohort and 0.972 in the temporal test cohort. The combination and nomogram models outperformed the clinical and single-sequence models.

**Conclusions:**

The combined radiomics model and nomogram showed improved performance in differentiating glioma recurrence from response to treatment compared with the single-sequence models. The nomogram showed discrimination comparable to that of the combined model and may provide additional clinical value for individualized assessment.

## Introduction

Gliomas are the most common primary intracranial tumors, accounting for about 27% of central nervous system tumors (1). The preferred clinical treatment is surgical resection and postoperative radiotherapy plus concurrent or adjuvant Temozolomide (TMZ) chemotherapy (2). It is important to note that gliomas are highly invasive. The growth of some gliomas continues despite radiotherapy and chemotherapy (3). Glioma is prone to recurrence and related post-treatment reactions (including radiation necrosis and postoperative pseudo-progression). Many studies have unified pseudo-progression and radiation necrosis as treatment responses (4, 5). The prognosis and survival of these two diseases are better than tumor recurrence and different from the treatment regimens after recurrence. Therefore, differentiating glioma recurrence from post-treatment reactions is of great clinical significance.

Conventional magnetic resonance sequences such as Contrast-enhanced T1 Weighted Imaging (CE-T1WI), T2 Weighted Imaging (T2WI) and T2 fluid-attenuated inversion recovery (T2-FLAIR) sequence were challenging to distinguish between the two outcomes. Conventional MRI (6–8) revealed significant heterogeneous enhancement in the corresponding areas, which was accompanied by large patches of surrounding edema (9).

A number of new MRI technologies are currently available to help differentiate glioma recurrence from response to treatment (10–13), such as Arterial Spin Labeling (ASL) technology, Amide Proton Transfer Weighted (APTW) imaging, Quantitative Susceptibility Mapping (QSM) and Diffusion Weighted Imaging (DWI). There is, however, a limitation to examining signal intensity differences in corresponding images. Radiomics can extract high-throughput quantitative image features from medical images (14). These features can not only reflect the differences in image shape and intensity but also reflect the spatial position relationship between different pixels, which is conducive to displaying tumor heterogeneity (15). The nomogram can be used to generate individual probabilities of clinical events by integrating other predictive variables, which is helpful in formulating the personalized diagnosis and treatment plans. In current research, nomograms are commonly used to combine tumors’ clinical and radiomics features to establish a more efficient prediction model (16, 17).

However, there is no report on radiomics or nomogram combining CE-T1WI, ASL, APTW, QSM and DWI to distinguish glioma recurrence from treatment response. Therefore, we sought to develop and validate a radiomics model or nomogram based on above MRI techniques for identifying glioma recurrence and treatment response.

## Materials and methods

### Patients

This study was approved by the institutional research committee of Shandong Provincial Hospital (SWYX: NO. 2022-565), and the requirement for informed consent was waived. From October 2019 to February 2024, patients with pathologically confirmed glioma who developed abnormal enhancement after radiotherapy and chemotherapy were retrospectively screened. Patients enrolled from October 2019 to June 2023 constituted the development cohort. After application of the predefined inclusion and exclusion criteria, 210 eligible patients were randomly divided at a 7:3 ratio into a training cohort (n=147) and a validation cohort (n=63). Patients enrolled during the subsequent non-overlapping period from July 2023 to February 2024 were screened using the same eligibility criteria and constituted an independent temporal test cohort (n=64). No patients enrolled during this later period met the exclusion criteria. The training cohort was used for model development, the validation cohort for internal validation, and the independent temporal test cohort for temporal validation. The patient-selection process is shown in **Figure 1**.

**Figure 1:**
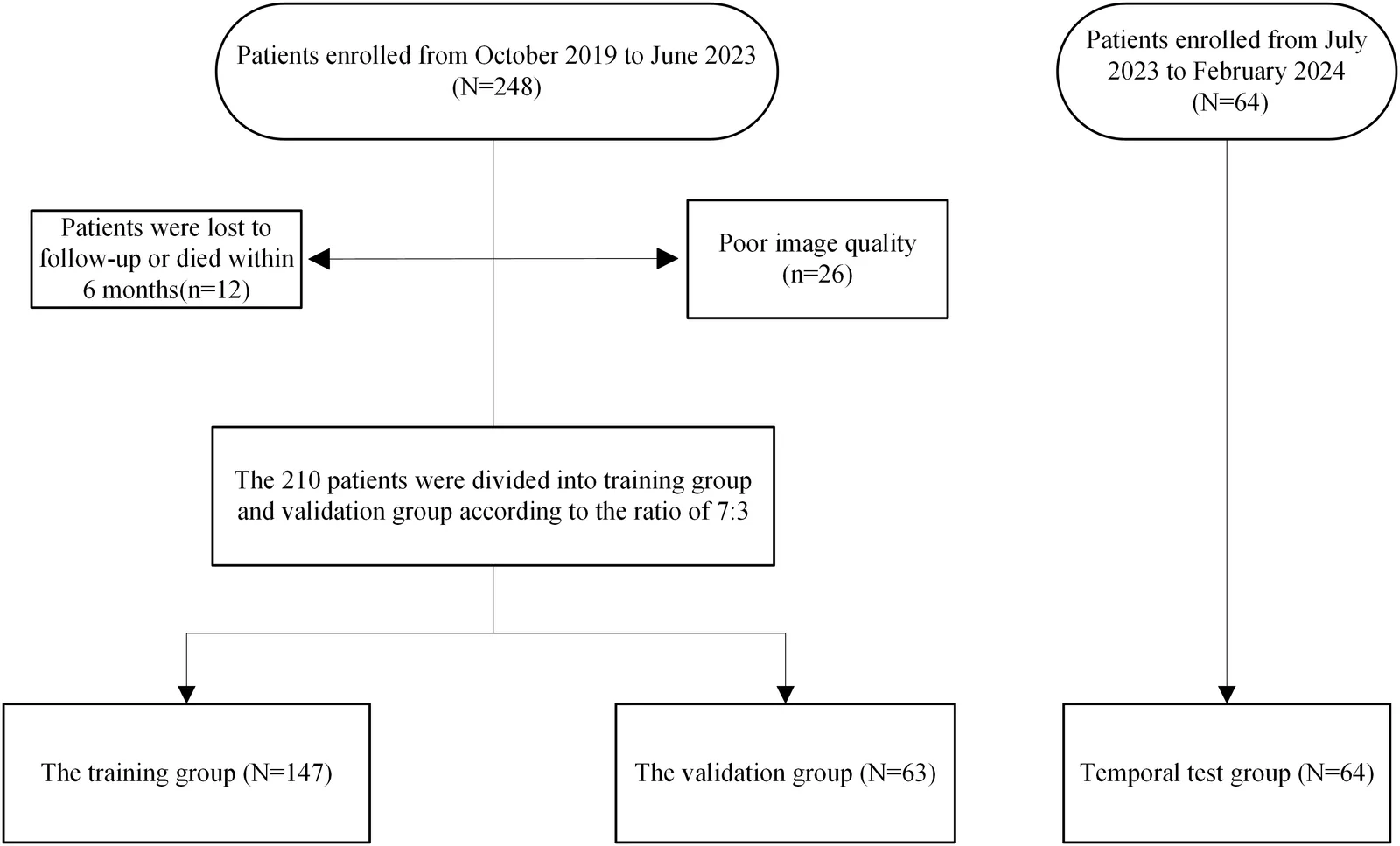
Flowchart of patient recruitment.

The inclusion criteria were as follows: (1) Patients underwent surgery in our hospital and were confirmed to have glioma by histopathological results; (2) Radiotherapy and chemotherapy were given after surgery; (3) Patients underwent conventional MRI and CE-T1WI at 24–48 hours after glioma surgery as baseline. (4) During the follow-up, imaging showed new or expanded enhancement lesions in the original lesion area. (5) Routine MRI, CE-T1WI, 3D-pCASL, 3D-APTW, 3D-GRE and DWI were performed during follow-up. (6) The new or expanded enhancement lesions during follow-up had histopathological examination or imaging follow-up results, the number of follow-up MRI examinations was not less than 2, and the imaging follow-up time was more than 6 months. Exclusion criteria were: (1) Patients were lost to follow-up or died within 6 months; (2) Images of insufficient quality or with severe artifacts were excluded.

The new or expanded enhancing lesions identified during follow-up were classified using either histopathological confirmation, when available, or the Response Assessment in Neuro-Oncology (RANO) criteria (18). The criteria for grouping the recurrence and post-treatment response cohorts are described in **Supplementary Material S1**. For all cases classified by imaging follow-up, longitudinal clinical and MRI data were independently reviewed according to the RANO criteria by two radiologists, each with more than 10 years of experience. A senior radiologist with more than 20 years of experience subsequently reviewed all imaging-follow-up cases and made the final reference-standard determination. Any disagreement between the two initial readers was resolved by the senior radiologist. The radiologists responsible for reference-standard adjudication were independent of the ROI segmentation, radiomics feature extraction, and model-development teams and were blinded to the radiomics results and model predictions. The CBF, APTW, QSM, and ADC images were not used for the RANO-based reference-standard adjudication. For pathology-confirmed cases, the reference standard was based on the final histopathological diagnosis reported by the Department of Pathology after biopsy or second surgery. The pathologists were blinded to the corresponding imaging findings, imaging-based classification, radiomics results, and model predictions, and were not involved in ROI segmentation, radiomics feature extraction, or model development.

The method of reference-standard verification (histopathology or RANO criteria) was recorded for each patient and was further summarized separately for the training, validation, and temporal test cohorts.

### Magnetic resonance imaging

MRI images were acquired with a Philips 3.0T MRI imaging system (Ingenia CX, Philips Healthcare, Best, the Netherlands). The head coil was a 32-channel coil. All patients underwent CE-T1WI, 3D-pCASL, 3D-APTW, 3D-GRE and DWI sequences. Specific parameters can be found in **Supplementary Material S2**.

### Selection of clinical factors and construction of clinical models

The clinical factors such as age, gender, isocitrate dehydrogenase 1 (IDH1) genotyping, tumor location and maximum tumor diameter of patients in the training, validation and temporal test cohorts were counted and compared. Variables with a univariate *P* value < 0.10 were considered candidates and entered into the multivariable logistic regression analysis. A *P* value < 0.05 in the multivariable analysis was considered statistically significant. Using the univariate analysis, clinical factors were included in the logistic regression model to establish the clinical model. Then in the multivariate analysis, statistically significant clinical factors (*P* < 0.05) were selected to construct the nomogram. Relative risk estimates for each clinical factor were expressed as odds ratios (OR) and their 95% confidence intervals.

### Image preprocessing and region of interest delineation

After image acquisition, all images were resampled to 1 × 1 × 1 mm³ and registered using CE-T1WI as the reference image. The ROIs were delineated on CE-T1WI and subsequently transferred to the registered ADC, QSM, CBF, and APTW maps. The ROIs encompassed the entire lesion, including cystic, necrotic, and hemorrhagic components. The whole-tumor volume was manually segmented slice by slice in three dimensions. Two radiologists with more than 8 years of experience independently performed ROI delineation. During segmentation and feature extraction, the radiologists were blinded to the final reference-standard classification (recurrence or treatment-related response), radiomics results, and model predictions. To assess feature reproducibility, 100 patients were randomly selected from the training cohort. One radiologist repeated the ROI delineation after a one-week interval for assessment of intra-observer reproducibility, while another radiologist independently delineated the same lesions for assessment of inter-observer reproducibility. Radiomics features were extracted from each segmentation. The same blinding procedure was maintained throughout the reproducibility assessment. Features with both intra-observer and inter-observer intraclass correlation coefficient (ICC) ≥ 0.80 were retained for subsequent analysis.

### Radiomics feature extraction and screening

Radiomics features were extracted from CE-T1WI, CBF, APTW, QSM, and ADC maps using the SlicerRadiomics extension in 3D Slicer (version 4.11.20210226), incorporating PyRadiomics version 3.0.1. Most radiomics feature definitions implemented in PyRadiomics are compliant with the feature definitions established by the Imaging Biomarker Standardization Initiative (IBSI), with documented implementation-specific differences. Before texture-feature calculation, gray-level intensities were discretized using a fixed bin width of 25, while a fixed bin count was not applied. All images had been resampled to an isotropic voxel size of 1 × 1 × 1 mm³ before feature extraction. A total of 851 features were extracted from each sequence, including 14 shape and size features, 18 first-order histogram features, 75 texture features, and 744 wavelet features, yielding a total of 4255 radiomics features across the five sequences. Radiomics features were normalized using Z-score transformation, with the mean and standard deviation calculated from the training cohort and subsequently applied to the validation and temporal test cohorts. All feature normalization parameters and feature-screening procedures were derived exclusively from the training cohort, and neither the validation cohort nor the temporal test cohort was used for feature selection.

In order to screen out irrelevant and redundant features, the Kolmogorov-Smirnov test was used to assess the normality of radiomics features in the training cohort. Normally distributed features were compared between the recurrence and treatment-response cohorts using the independent-samples t-test, whereas non-normally distributed features were compared using the Mann-Whitney U test. To control for multiple comparisons, the Benjamini-Hochberg false-discovery-rate (FDR) correction was applied to the resulting *P* values. Features with an FDR-adjusted *P* value <0.05 were retained. The retained features were subsequently subjected to least absolute shrinkage and selection operator (LASSO) regression in the training cohort for further dimensionality reduction and feature selection. The penalty parameter (λ) was determined using 10-fold cross-validation, and the value corresponding to the minimum cross-validation error (lambda.min) was selected. Features with non-zero coefficients at lambda.min were retained for subsequent model construction. The LASSO regression algorithm can reduce the regression coefficient of features by penalizing the absolute value of the regression coefficient to remove unimportant features and reduce the weight of extreme features (19). **Figure 2** depicted the flow chart of this study.

**Figure 2:**
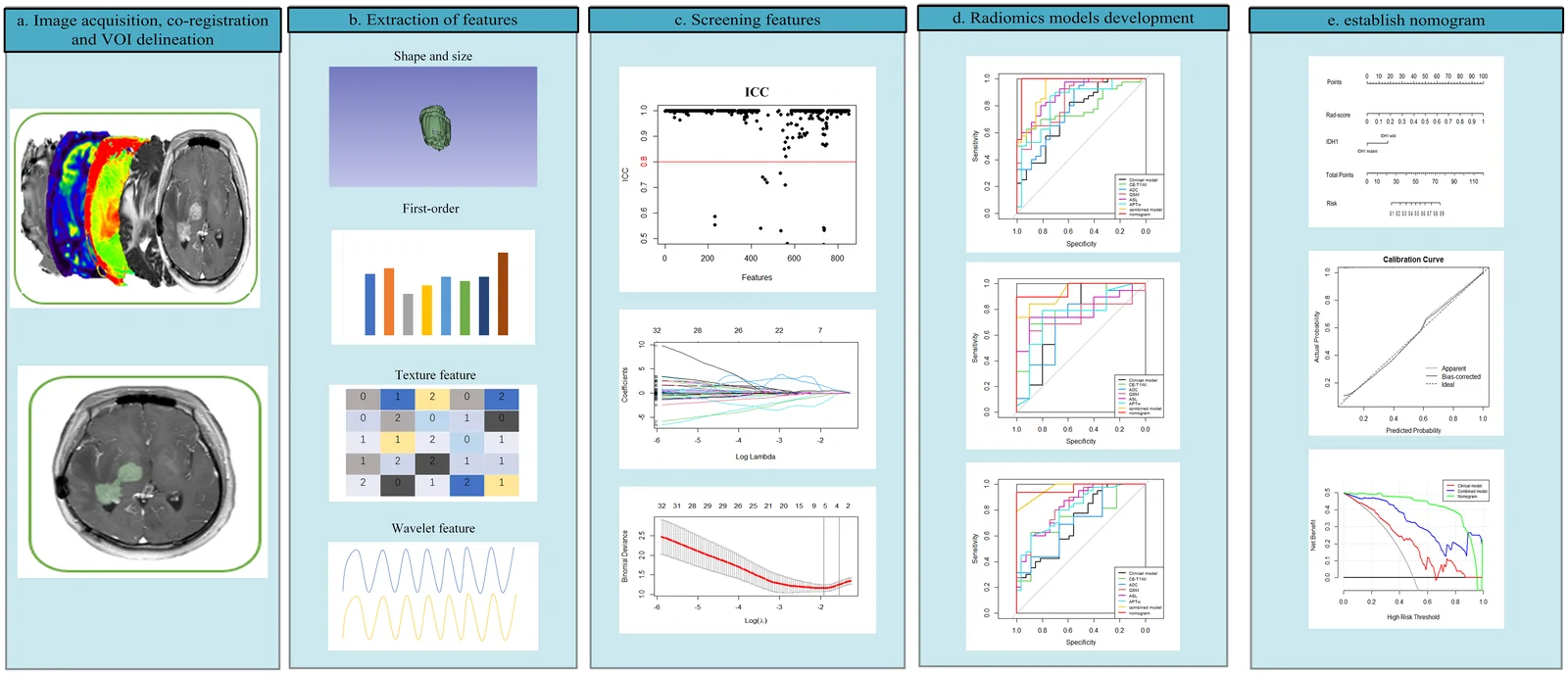
Flow chart of this study. CE-T1WI, ADC map, QSM, APTW and CBF map images were acquired and registered and delineated the region of interest **(a)**. Extract shape and size features, first-order histogram features, texture features and wavelet features **(b)**. Features with an intraclass correlation coefficient ≥ 0.80 were retained and further screened using LASSO regression. The coefficient profile and cross-validation curve for selection of the optimal penalty parameter (λ) are shown, with lambda.min used for feature selection. **(c)**. The radiomics model was established and evaluated by ROC curve and AUC **(d)**. Build a nomogram and evaluate the model **(e)**.

### Establishment and validation of radiomics model

Five single-sequence radiomics models and one combined model were constructed based on the above-selected radiomics features in conjunction with a binary logistic regression model. The Receiver Operator Characteristic (ROC) Curve of each model was drawn and the Area Under the Curve (AUC), sensitivity, specificity and accuracy were calculated. DeLong test was applied to compare the difference of AUC between each model to determine the best performance model. There was a statistically significant difference in AUC between the two models at *P* < 0.05.

### Development and validation of nomograms

Based on the selected features in the model, the radiomics score (Rad-score) was calculated for each patient. The nomogram was constructed by combining the radiomics score of each patient and the clinical chosen factors in the multivariate analysis. The ROC curve of the nomogram was drawn and its AUC, sensitivity, specificity and accuracy were calculated. The DeLong test was used to compare AUCs between models within each cohort. *P* < 0.05 was considered as statistically significant. The Hosmer-Lemeshow test and Decision Curve Analysis (DCA) were used to evaluate the value of the nomogram.

### Statistical analysis

SPSS statistical software (Ver.22.0) and R language statistical software (Ver.4.1.1) were used for statistical analysis. Measurement data were presented as mean ± standard deviation (x ± s). Categorical data were presented as frequency (%). The χ² test or Fisher exact test was used for categorical variables. A *P* value of less than 0.05 was considered statistically significant. The “caret” package in R language software was used to normalize the feature data. Lasso regression algorithm was performed using the “glmnet” package. The ROC curves of each model were plotted using the “pROC” package. “Formula”, “ggplot2”, “rms” and “rmda” software packages were used to draw nomograms, calibration curves and DCA. This study was reported in accordance with the CheckList for EvaluAtion of Radiomics research (CLEAR) guideline, and the completed checklist is provided in **Supplementary Material S3** (20).

## Results

### Patient characteristics

Finally, 274 patients were enrolled in this study and 38 patients were excluded. Among the eligible patients enrolled from October 2019 to June 2023, 210 were randomly divided at a 7:3 ratio into the training cohort (n=147; 97 recurrence and 50 treatment-response cases) and validation cohort (n=63; 41 recurrence and 22 treatment-response cases). An additional 64 eligible patients enrolled consecutively from July 2023 to February 2024 constituted the independent temporal test cohort, including 44 recurrence and 20 treatment-response cases. Training set data were primarily used for model construction, while validation and temporal test set data were used to verify the model’s performance.

Of the 182 patients classified as having recurrence, 67 were histopathologically confirmed and 115 were classified by RANO criteria. Among the 92 patients classified as having treatment-related response, 10 were histopathologically confirmed and 82 were classified by RANO criteria. The reference-standard verification methods were further stratified by cohort. In the training cohort, 36 of 97 recurrence cases and 6 of 50 treatment-response cases were histopathologically confirmed, whereas 61 and 44, respectively, were classified by RANO criteria. In the validation cohort, 15 of 41 recurrence cases and 2 of 22 treatment-response cases were histopathologically confirmed, whereas 26 and 20, respectively, were classified by RANO criteria. In the temporal test cohort, 16 of 44 recurrence cases and 2 of 20 treatment-response cases were histopathologically confirmed, whereas 28 and 18, respectively, were classified by RANO criteria. The overall proportions of histopathological verification were comparable across the training, validation, and temporal test cohorts (28.6%, 27.0%, and 28.1%, respectively). Detailed information is provided in **Supplementary Material S4**. **Figures 3** and **4** showed patient images of partial recurrences versus post-treatment responses.

**Figure 3:**
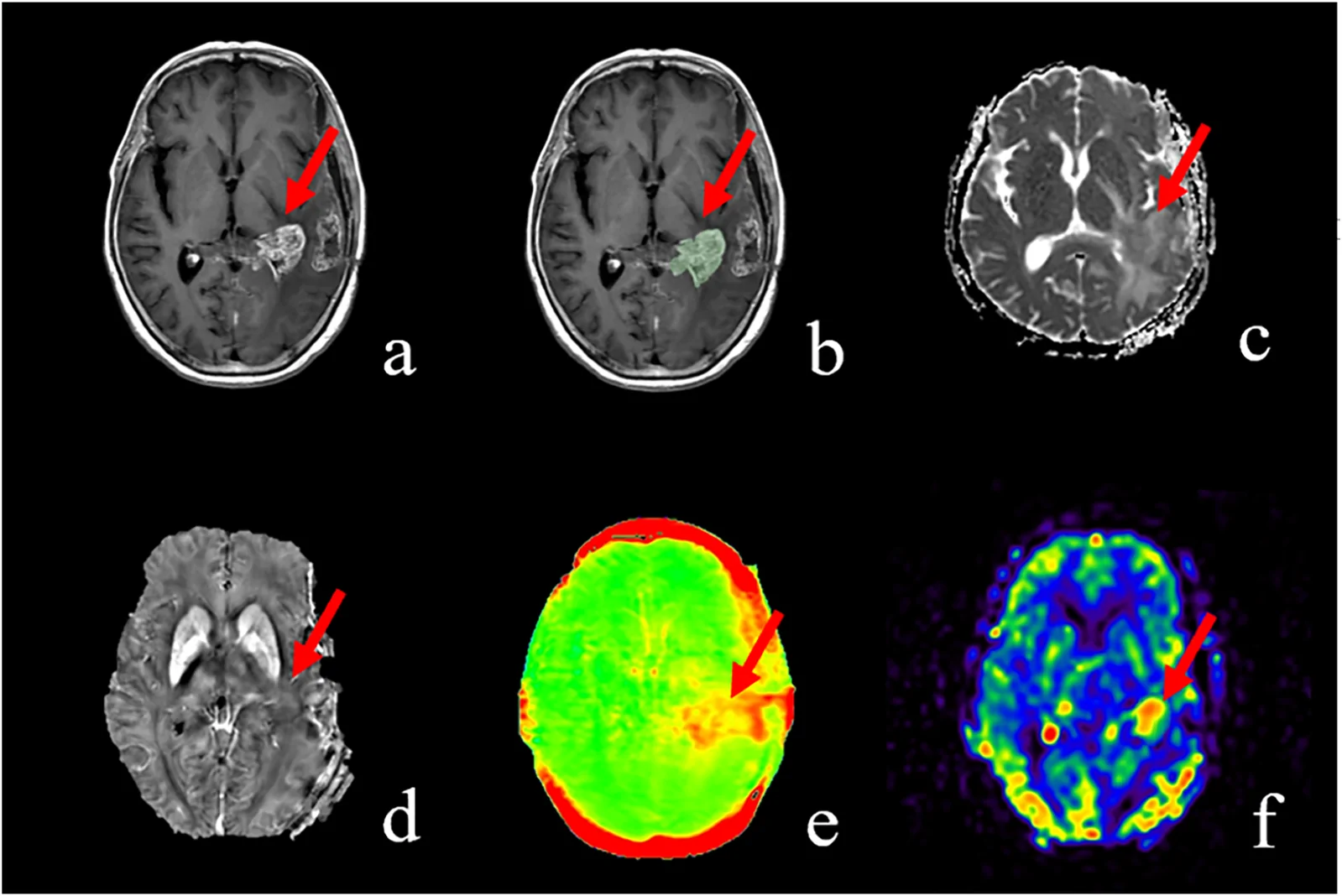
Recurrent cases of glioma after surgery. A 64-year-old woman presented with left lateral ventricle postoperative recurrence. The red arrow points to the recurrent lesion. CE-T1WI image **(a)**. ROI delineation **(b)**. ADC map **(c)**. QSM images **(d)**. APTW pseudo-color image **(e)**. CBF pseudo-color map**(f)**.

**Figure 4:**
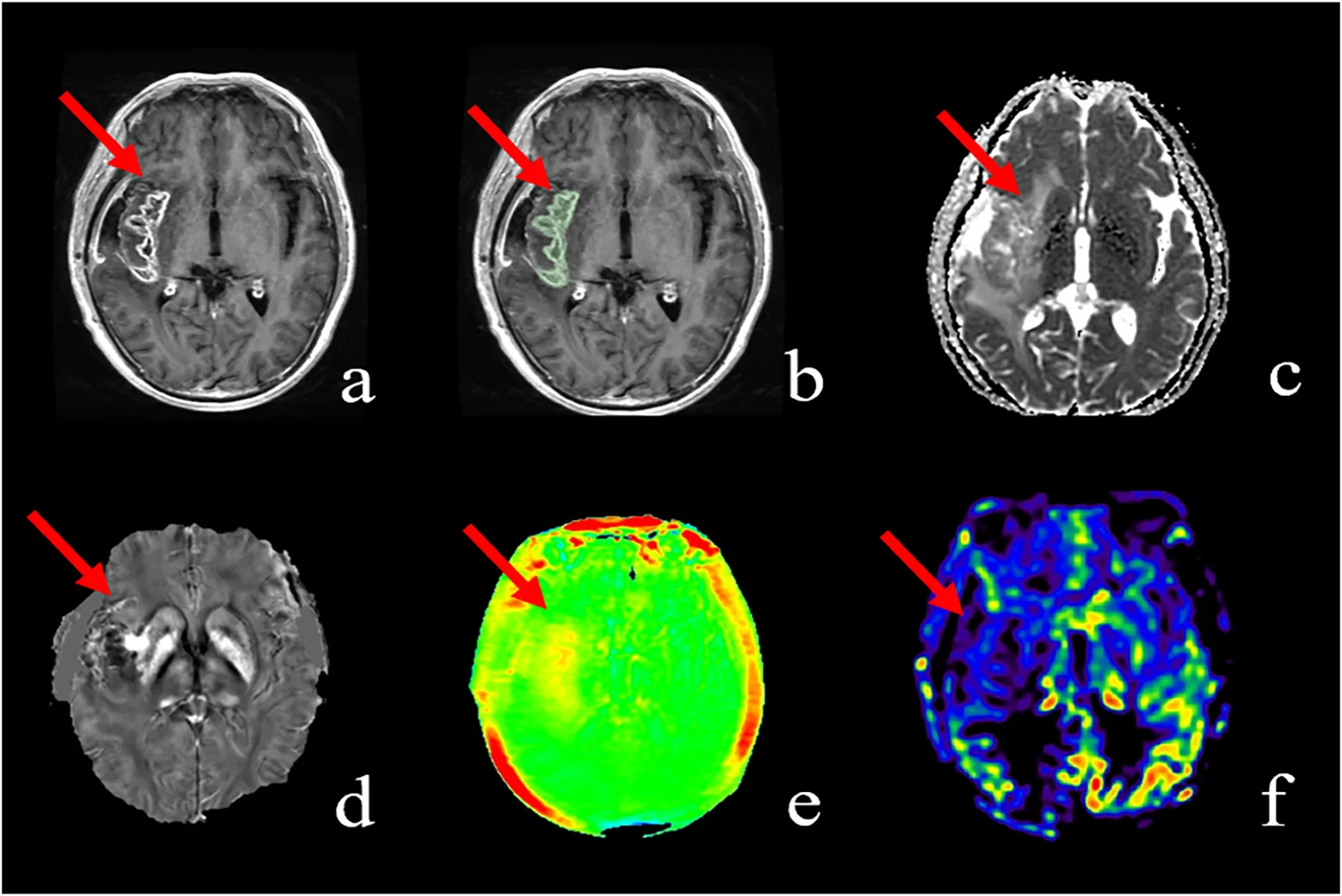
Cases of post-treatment response to glioma. After treatment, a 61-year-old man presented with the right temporal lobe as the response area. The red arrow indicates the post-treatment response area. CE-T1WI image **(a)**. ROI delineation **(b)**. ADC map **(c)**. QSM images **(d)**. APTW pseudo-color image **(e)**. CBF pseudo-color map **(f)**.

### Selection of clinical factors and efficacy of clinical models

**Table 1** shows the differences in clinical factors between patients in the training, validation and temporal test cohorts. The training cohort showed significant differences in age, maximum tumor diameter, and IDH1 genotype (*P* < 0.05). There was no significant difference in gender and tumor location (*P* > 0.05). **Table 2** shows the results of univariate and multivariate analyses. In the univariate analysis, age, maximum tumor diameter, and IDH1 genotype met the prespecified screening criterion (*P* < 0.10) and were therefore entered into the multivariable logistic regression analysis to construct the clinical model. Gender and tumor location did not meet this criterion and were not included in the multivariable model. In the training cohort, the AUC of the clinical model was 0.748, the sensitivity was 0.850, the specificity was 0.444, and the accuracy was 0.687. In the validation cohort, the AUC of the clinical model was 0.721, the sensitivity was 0.684, the specificity was 0.500, and the accuracy was 0.621. In the temporal test cohort, the AUC of the clinical model was 0.706, the sensitivity was 0.800, the specificity was 0.444, and the accuracy was 0.657. According to the multivariate analysis, the IDH1 genotype was statistically significant in both the recurrence and response cohorts (*P* < 0.05). The IDH1 genotyping was thus selected for inclusion in the nomogram construction.

**Table 1:** Clinical information of enrolled patients.

Clinical factors	Training cohort		*P* value	Validation cohort		*P* value	Temporal test cohort		*P* value
	Recurrence (n=97)	Treatment response (n=50)		Recurrence (n=41)	Treatment response (n=22)		Recurrence (n=44)	Treatment response (n=20)	
Age	57.1 ± 9.2	46.4 ± 11.3	0.008*	51.1 ± 11.1	52.3 ± 13.7	0.839	54.1 ± 9.1	51.3 ± 13.7	0.825
Maximum tumor diameter (cm)	6.34 ± 1.26	4.41 ± 1.27	0.005*	4.14 ± 1.12	4.11 ± 1.16	0.983	4.01 ± 1.19	4.25 ± 1.14	0.855
Gender			0.978			0.914			0.932
male	52(53.61%)	27(54.00%)		22(53.66%)	12(54.55%)		18(40.91%)	9(45.00%)	
female	45(46.39%)	23(46.00%)		19(46.34%)	10(45.46%)		26(59.09%)	11(55.00%)	
IDH1 genotype			0.003*			0.058			0.053
Wild type	75(77.32%)	26(52.00%)		27(65.85%)	17(77.27%)		30(68.18%)	15(75.00%)	
Mutant type	22(22.68%)	24(48.00%)		14(34.15%)	5(22.73%)		14(31.82%)	5(25.00%)	
Location of the tumor			0.431			0.682			0.322
Frontal lobe	23(23.71%)	9(18.00%)		9(21.95%)	7(31.82%)		10(22.73%)	7(35.00%)	
Parietal lobe	25(25.77%)	6(12.00%)		11(26.83%)	2(9.09%)		12(27.27%)	1(5.00%)	
Temporal lobe	23(23.71%)	15(30.00%)		14(34.15%)	8(36.36%)		14(31.82%)	5(25.00%)	
Occipital lobe	16(16.49%)	12(24.00%)		4(9.76%)	4(18.18%)		4(9.09%)	4(20.00%)	
Other locations	10(10.31%)	8(16.00%)		3(7.32%)	1(4.55%)		4(9.09%)	3(15.00%)	

An asterisk (*) indicates that the data are statistically different between the recurrence cohort and the post-treatment response cohort (*P* < 0.05).

**Table 2:** Results of univariate analysis and multivariate analysis of clinical factors.

	Univariate analysis		Multivariate analysis	
Clinical factors	OR (95% CI)	*P* value	OR (95% CI)	*P* value
Age	1.129(1.002~1.256)	0.026*	1.469(0.843~1.672)	0.765
Gender	0.882(0.355~1.812)	0.539		
Maximum tumor diameter (cm)	1.323(1.137~1.540)	0.017*	1.742(0.475~2.357)	0.436
IDH1 genotype	3.885(2.311~7.431)	0.014*	5.353(1.235~21.976)	0.021**
Location of the tumor	0.797(0.567~1.467)	0.587		

An asterisk (*) indicates variables meeting the prespecified univariate screening criterion for entry into the multivariable analysis (*P* < 0.10), whereas a double asterisk (**) indicates variables that remained statistically significant in the multivariable analysis (*P* < 0.05). Blank cells in the multivariable columns indicate variables that were not entered into the multivariable model because they did not meet the univariate screening criterion. Odds ratios (95%) represent odds ratios and 95% confidence intervals. IDH1 is isocitrate dehydrogenase 1.

### Results of feature screening

After retaining features with both intra-observer and inter-observer ICCs ≥ 0.80, univariate comparisons followed by Benjamini-Hochberg FDR correction (FDR-adjusted *P* < 0.05) retained 152 features from CE-T1WI, 264 from the CBF maps, 281 from the APTW maps, 232 from the QSM maps, and 174 from the ADC maps. A total of 1,103 candidate features were subsequently subjected to LASSO regression in the training cohort. Finally, 2 radiomics features were retained for the CE-T1WI model, 4 for the CBF model, 5 for the APTW model, 4 for the QSM model, 3 for the ADC model, and 6 for the combined model. The six features included in the combined model comprised two CBF features, two APTW features, one QSM feature, and one ADC feature. The selected radiomics features are shown in **Table 3**.

**Table 3:** Radiomics features of single sequence model and combination model.

Models	Features number	Name of feature
Combined model	6	CBF-waveletHLH-glszm-ZoneEntropy
		CBF-original-gldm-DependenceVariance
		APTW-waveletLHL-firstorder-Mean
		APTW-waveletLHH-glcm-Imc1
		QSM-waveletHHL-glszm-ZoneVariance
		ADC-original-firstorder-RootMeanSquared
CE-T1WI model	2	CE-T1WI-waveletHLH-glcm-ClusterShade
		CE-T1WI-waveletHLH- glszm-ZoneEntropy
CBF map model	4	CBF-waveletHLL-glrlm-ShortRunEmphasis
		CBF-waveletHLH-glszm-ZoneEntropy
		CBF-original-gldm-DependenceVariance
		CBF- waveletLHL-glszm-ZoneVariance
APTW model	5	APTW-waveletLHL-firstorder-Mean
		APTW- waveletLLH-glszm-LargeAreaEmphasis
		APTW-waveletLHH-glcm-Imc1
		APTW- waveletHLL-glszm-ZoneVariance
		APTW- original-gldm-DependenceEntropy
QSM model	4	QSM-waveletHHL-glszm-ZoneVariance
		QSM-waveletLLL-firstorder-Maximum
		QSM- waveletLHL-glcm-Correlation
		QSM- waveletLLH-glszm-LargeAreaEmphasis
ADC map model	3	ADC- waveletHHH-glcm-InverseVariance
		ADC-original-firstorder-RootMeanSquared
		ADC- waveletHHH-glcm-DifferenceAverage

### Performance and comparison of radiomics models

The AUC for the five single-sequence models ranged from 0.781-0.899 in the training cohort, from 0.705-0.789 in the validation cohort and from 0.708-0.828 in temporal test cohort. The AUC of the combined model was 0.927, 0.934 and 0.968 in the training cohort, validation cohort and temporal test cohort, respectively. In both training, validation and temporal test cohorts, the combined model had the largest AUC and the best diagnostic performance among the 6 radiomics models (5 single-sequence models and 1 combined model). After DeLong test, the differences in AUC between the combined model and the other five single-sequence radiomics models were statistically significant (*P* < 0.05). The diagnostic efficacy of each model is shown in **Table 4**, and the results of DeLong test are shown in **Table 5**. The ROC curves of the training, validation and temporal test cohorts and of each model are shown in **Figure 5**.

**Table 4:** Diagnostic efficacy of clinical model, radiomics model and nomogram.

Models	Cohort	AUC (95% CI)	Sensitivity (95% CI)	Specificity (95% CI)	Accuracy (95% CI)
Clinical model	Training cohort	0.748(0.627~0.870)	0.850 (0.766–0.908)	0.444 (0.315–0.581)	0.687 (0.608–0.756)
	Validation cohort	0.721(0.493~0.949)	0.684 (0.531–0.805)	0.500 (0.307–0.693)	0.621 (0.498–0.731)
	Temporal test cohort	0.706(0.578~0.835)	0.800 (0.660–0.892)	0.444 (0.253–0.653)	0.657 (0.535–0.761)
CE-T1WI model	Training cohort	0.781(0.672~0.891)	0.725 (0.629–0.804)	0.481 (0.349–0.616)	0.627 (0.547–0.701)
	Validation cohort	0.789(0.616~0.963)	0.789 (0.642–0.886)	0.700 (0.491–0.849)	0.759 (0.641–0.848)
	Temporal test cohort	0.729(0.520~0.938)	0.750 (0.606–0.854)	0.667 (0.449–0.831)	0.720 (0.600–0.815)
ADC map model	Training cohort	0.787(0.674~0.900)	0.875 (0.795–0.927)	0.556 (0.419–0.685)	0.746 (0.670–0.809)
	Validation cohort	0.705(0.486~0.925)	0.734 (0.583–0.845)	0.600 (0.396–0.775)	0.690 (0.568–0.790)
	Temporal test cohort	0.708(0.491~0.926)	0.750 (0.606–0.854)	0.556 (0.347–0.747)	0.680 (0.558–0.781)
CBF map model	Training cohort	0.899(0.828~0.970)	0.800 (0.710–0.867)	0.778 (0.645–0.871)	0.791 (0.718–0.849)
	Validation cohort	0.789(0.621~0.958)	0.895 (0.765–0.957)	0.602 (0.398–0.776)	0.780 (0.663–0.865)
	Temporal test cohort	0.824(0.723~0.926)	0.925 (0.809–0.973)	0.519 (0.315–0.717)	0.761 (0.644–0.849)
QSM model	Training cohort	0.843(0.748~0.937)	0.925 (0.855–0.963)	0.593 (0.455–0.718)	0.791 (0.718–0.849)
	Validation cohort	0.747(0.567~0.982)	0.842 (0.702–0.923)	0.500 (0.307–0.693)	0.724 (0.603–0.819)
	Temporal test cohort	0.829(0.730~0.927)	0.900 (0.777–0.959)	0.519 (0.315–0.717)	0.746 (0.628–0.837)
APTW model	Training cohort	0.825(0.719~0.931)	0.925 (0.855–0.963)	0.593 (0.455–0.718)	0.791 (0.718–0.849)
	Validation cohort	0.750(0.567~0.928)	0.895 (0.765–0.957)	0.414 (0.236–0.617)	0.690 (0.568–0.790)
	Temporal test cohort	0.809(0.730~0.927)	0.925 (0.809–0.973)	0.481 (0.283–0.685)	0.746 (0.628–0.837)
Combined model	Training cohort	0.927(0.864~0.990)	0.925 (0.855–0.963)	0.778 (0.645–0.871)	0.866 (0.802–0.912)
	Validation cohort	0.934(0.851~1.000)	0.842 (0.702–0.923)	0.700 (0.491–0.849)	0.793 (0.678–0.875)
	Temporal test cohort	0.968(0.926~1.000)	1.000 (0.920–1.000)	0.700 (0.481–0.855)	0.897 (0.799–0.950)
Nomogram	Training cohort	0.983(0.950~1.000)	0.975 (0.921–0.992)	0.963 (0.870–0.990)	0.970 (0.928–0.988)
	Validation cohort	0.958(0.892~1.000)	0.895 (0.765–0.957)	1.000 (0.851–1.000)	0.931 (0.841–0.972)
	Temporal test cohort	0.972(0.914~1.000)	0.938 (0.826–0.980)	1.000 (0.839–1.000)	0.960 (0.881–0.987)

AUC (95%CI) represents the area under the receiver operating characteristic curve and its 95% confidence interval.

**Table 5:** Comparison results of AUC between nomogram and radiomics models.

Delong Test	Cohort	Combined model	Nomogram
Clinical model	Training cohort	*P*=0.007	*P*=0.003
	Validation cohort	*P*=0.027	*P*=0.021
	Temporal test cohort	*P*=0.018	*P*=0.014
CE-T1WI model	Training cohort	*P*=0.032	*P*<0.001
	Validation cohort	*P*=0.027	*P*=0.047
	Temporal test cohort	*P*=0.022	*P*=0.012
ADC map model	Training cohort	*P*=0.017	*P*=0.005
	Validation cohort	*P*=0.026	*P*=0.023
	Temporal test cohort	*P*=0.015	*P*=0.009
CBF map model	Training cohort	*P*=0.022	*P*=0.022
	Validation cohort	*P*=0.028	*P*=0.025
	Temporal test cohort	*P*=0.026	*P*=0.024
QSM model	Training cohort	*P*=0.014	*P*=0.003
	Validation cohort	*P*=0.047	*P*=0.035
	Temporal test cohort	*P*=0.034	*P*=0.017
APTW model	Training cohort	*P*=0.012	*P*=0.004
	Validation cohort	*P*=0.033	*P*=0.027
	Temporal test cohort	*P*=0.030	*P*=0.020
Combined model	Training cohort		*P*=0.411
	Validation cohort		*P*=0.721
	Temporal test cohort		*P*=0.521

**Figure 5:**
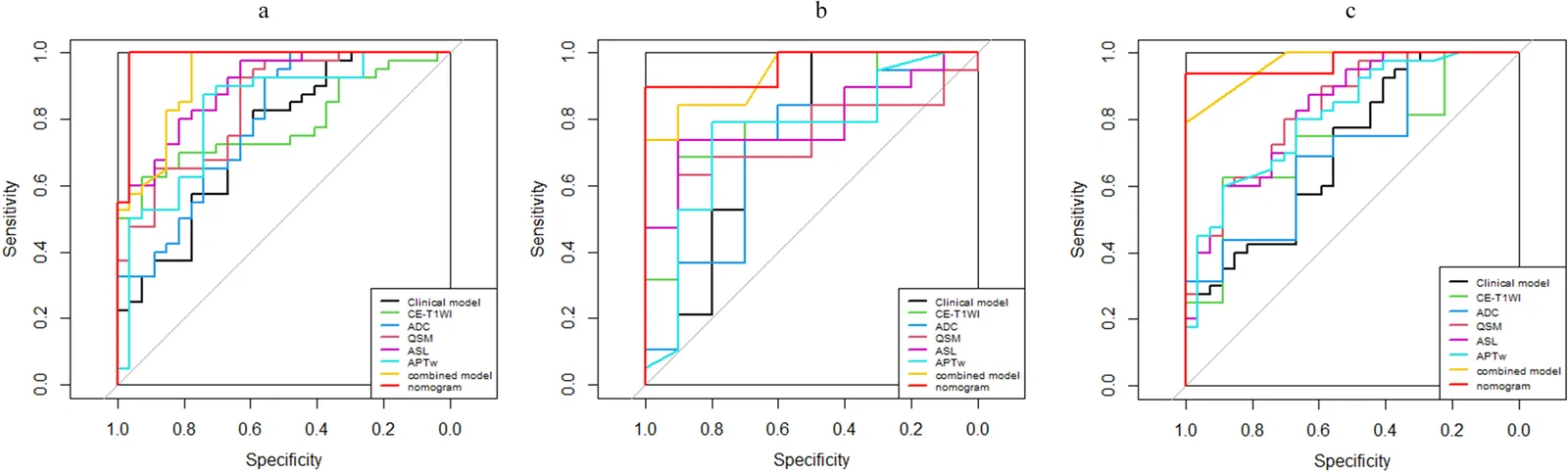
ROC curves of the training, validation and temporal test cohorts for each model. ROC curves of the training cohort **(a)**. ROC curves of the validation cohort **(b)**. ROC curves of the temporal test cohort **(c)**.

### Diagnostic efficacy of the nomogram

The radiomics score was calculated for each patient in the combined model (Rad-score) based on the features included in the combined model. Specific radiomics scores were calculated using the following formula:

Rad-score=2.819×APTW-waveletLHL-first-order-Mean+1.316×APTW-waveletLHH-glcm-Imc1 + 5.285×CBF-waveletHLH-glszm-ZoneEntropy+2.225×CBF-original-gldm-DependenceVariance-2.197×QSM-waveletHHL-glszm-ZoneVariance-1.020×ADC-original-firstorder-RootMeanSquared+4.846.

The radiomics score and IDH1 genotyping of the training cohort were used to construct the nomogram. The nomogram is shown in **Figure 6**. In the nomogram model, the AUC of the training cohort was 0.983, the sensitivity was 0.975, the specificity was 0.963, and the accuracy was 0.970. The AUC of the validation cohort was 0.958, the sensitivity was 0.895, the specificity was 1.000, and the accuracy was 0.931. The AUC of the temporal test cohort was 0.972, the sensitivity was 0.938, the specificity was 1.000, and the accuracy was 0.960. After DeLong test, the differences in AUC between the nomogram and the other five single sequence models and clinical models were statistically significant (*P* < 0.05), and there was no significant difference in AUC between the nomogram and the combination model (*P* > 0.05) in both training, validation and temporal test cohorts. The results are shown in **Table 5**. The calibration curve of the nomogram indicated a good agreement between the predicted and actual probabilities, and the results are shown in **Figure 7**. As the result of the Hosmer-Lemeshow test, the *P* values were 0.585 in the training cohort, 0.654 in the validation cohort and 0.535 in the temporal test cohort, respectively, indicating that the model was well fitted. The DCA curves of the clinical model, the combined model and the nomogram in the validation cohort showed the net benefit of the nomogram was the highest, clinical model was the lowest, and combined model was medium over a wide range of threshold probabilities (0-95%). The results of the DCA curve are shown in **Figure 8**.

**Figure 6:**
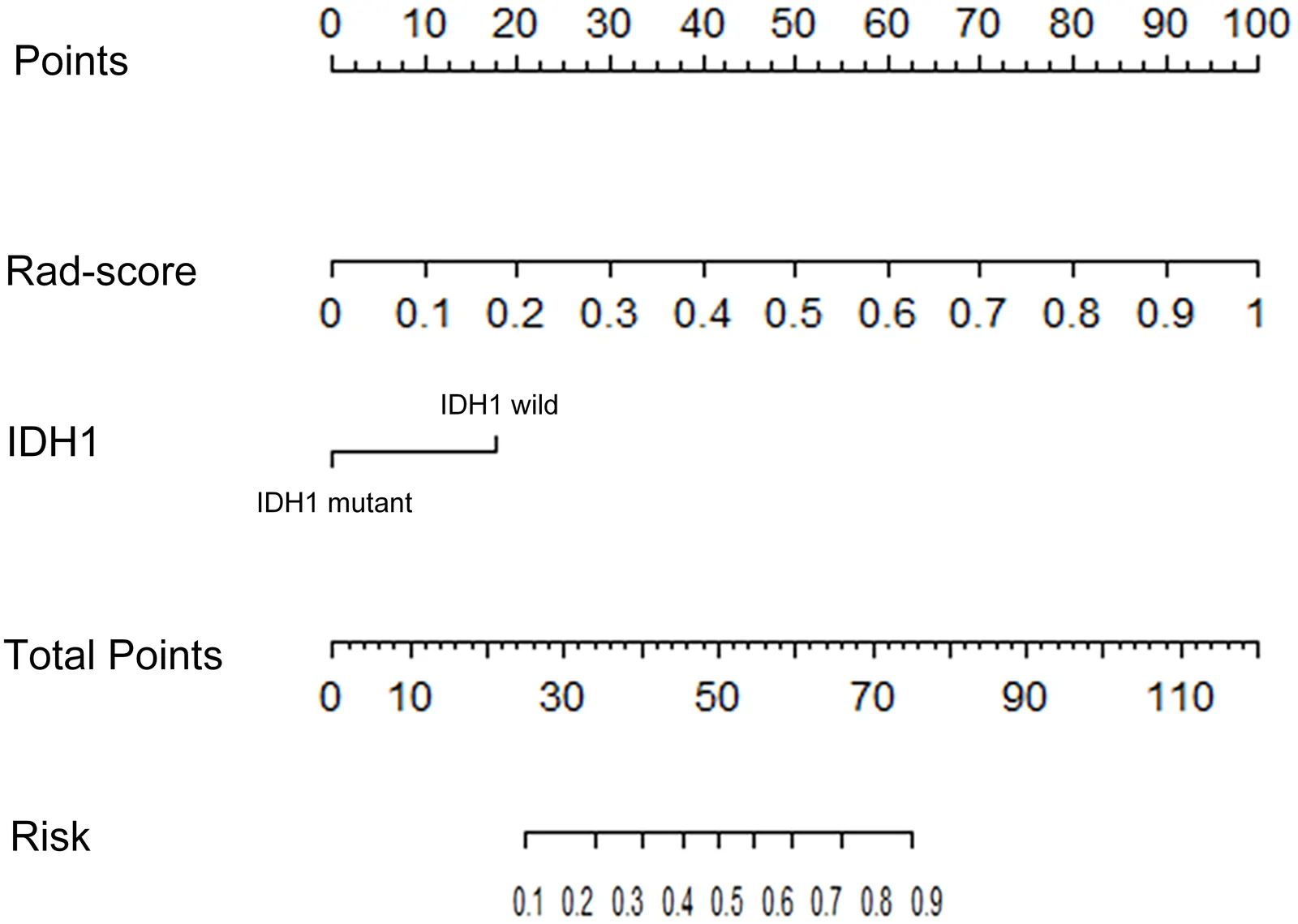
Nomogram integrating the radiomics score (Rad-score) and IDH1 genotype for differentiating glioma recurrence from treatment-related response.

**Figure 7:**
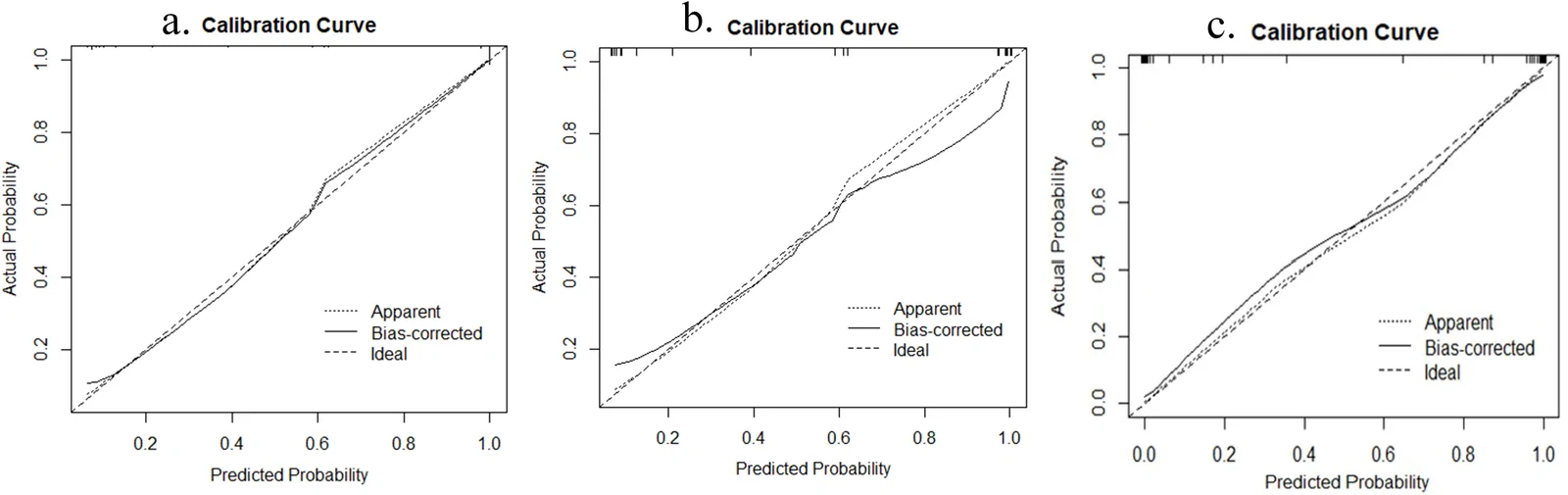
Calibration curves of the nomogram training, validation and temporal test cohorts. The calibration curve of the training cohort **(a)**. The calibration curve of the validation cohort **(b)**. The calibration curve of the temporal test cohort **(c)**. The horizontal axis is the model's predicted probability, the vertical axis is the actual probability of the model, the solid line is the calibration curve represented by the prediction model, and the dashed diagonal line is the perfect fitting curve in an ideal state. This study’s solid line in the prediction model is close to the dashed diagonal line, indicating that the predicted and observed probabilities showed good agreement.

**Figure 8:**
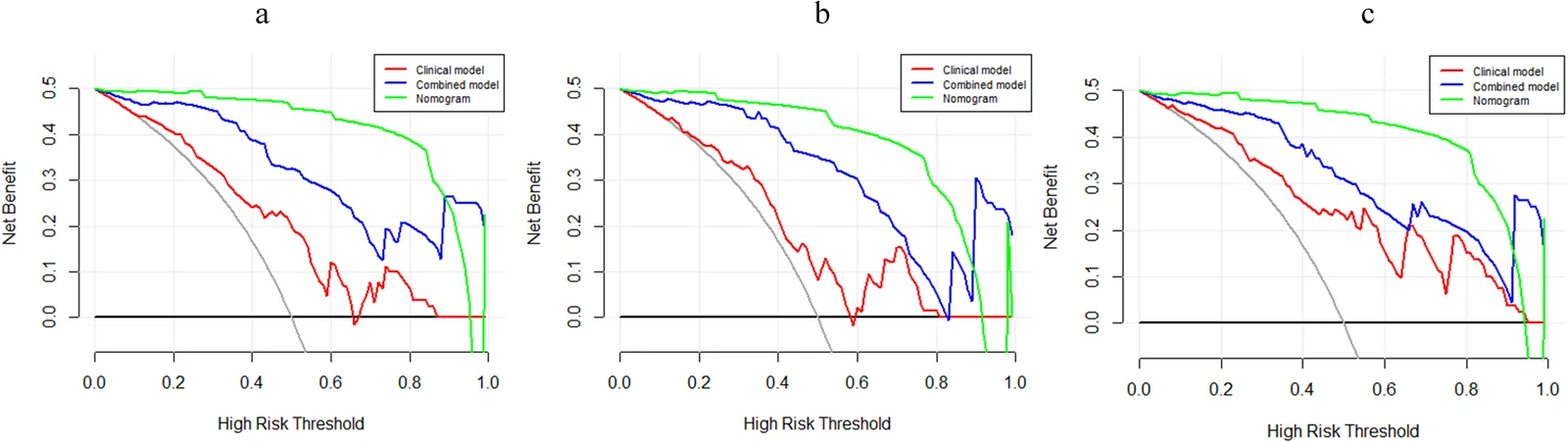
Decision curve analysis of the nomogram. The DCA curve of the training cohort **(a)**. The DCA curve of the validation cohort **(b)**. The DCA curve of the temporal test cohort **(c)**. It is noted that red lines represent clinical models, blue lines represent combined models, and green lines represent nomograms. The X-axis represents the high-risk threshold and the Y-axis represents the net benefit. The higher the curve, the greater the net benefit, and the higher the clinical value of the corresponding model.

## Discussion

This study constructed a clinical model, 6 radiomics models and a nomogram model to identify glioma recurrence and response to treatment. There is good discrimination between glioma recurrence and response to treatment using both the combination model and the nomogram. Although the nomogram did not significantly improve discrimination compared with the combined radiomics model, DCA suggested a potential additional clinical benefit, supporting its value for individualized clinical assessment.

A clinical model was constructed based on age, tumor diameter, and IDH1 genotype. Although age and maximum tumor diameter were significant in the univariate analysis, their associations were substantially attenuated after multivariable adjustment, whereas IDH1 genotype remained significant. This may reflect overlapping explanatory information or partial intercorrelation among these clinical variables, resulting in limited independent predictive contributions of age and tumor diameter after adjustment. The AUC of the clinical model was 0.748 in the training cohort, 0.721 in the validation cohort, and 0.706 in the temporal test cohort. The diagnostic performance was moderate, indicating that clinical factors alone provided limited discriminatory information. Notably, age, maximum tumor diameter, and IDH1 genotype showed significant between-group differences in the training cohort but not in the validation or temporal test cohorts. This variability may partly reflect the smaller sample sizes and lower statistical power of the validation and temporal cohorts, together with sampling variability and differences in case mix. This instability may also partly explain the relatively low specificity of the clinical model across cohorts. In particular, IDH1 genotype remained close to statistical significance in both the validation (*P* = 0.058) and temporal test (*P* = 0.053) cohorts. This cohort-to-cohort variability may also partly explain the lower performance of the clinical model in the validation and temporal test cohorts. Radiomics, in contrast, can provide richer quantitative information from the images (21).

This study did not include CE-T1WI sequence features in the final combination model, following selection by LASSO regression algorithm. Five single-sequence image-based radiomics models were constructed in this study, and all five models were able to distinguish postoperative recurrence and response to treatment. The AUC of the single-sequence radiomics model ranged from 0.781-0.899 in the training cohort, from 0.705-0.789 in the validation cohort and from 0.708-0.828 in temporal test cohort. This is because of the differences in the cellular density, the proliferation of micro-vessels, the activity of protein synthesis, and the local focus of hemorrhage between areas of recurrence and areas of response after treatment (22–24). ADC map, CBF map, APTW map and QSM directly or indirectly showed the degree of cell density, the degree of microvascular proliferation, the active degree of protein synthesis and the local microhemorrhage. Recent radiomics research has further demonstrated that the addition of ADC and CBF features can provide incremental information beyond structural MRI for glioma molecular characterization, supporting the complementary value of diffusion- and perfusion-derived quantitative imaging features (25).

To comprehensively evaluate the heterogeneity of tumors, it is necessary to combine the comprehensive information of structural and functional changes (26). The combination of multiple sequences of magnetic resonance images can improve the diagnostic efficiency of the model. Ren et al. developed multimodality MRI radiomics models to differentiate true tumor recurrence from treatment-related effects in postoperative glioma and demonstrated that the integration of multiple MRI sequences provided improved diagnostic performance compared with single-sequence models (27). Other studies have added diffusion-weighted and perfusion-weighted imaging to the conventional MRI radiomics model. The results show that the multi-sequence combination model’s diagnostic performance is better than any single-sequence model (28). This study selected the radiomics features from CBF map, APTW, QSM, and ADC map to construct a combined model. Notably, no CE-T1WI features were retained in the final combined model, suggesting that the quantitative information provided by CBF, APTW, QSM, and ADC may contribute greater complementary value for this classification task. However, this finding should not be interpreted as indicating that CE-T1WI can be omitted from routine clinical imaging protocols, particularly because it remains essential for lesion assessment and ROI delineation. The combined model had high diagnostic efficiency, with an AUC of 0.927 in the training cohort, 0.934 in the validation cohort and 0.968 in temporal test cohort. Based on the DeLong test, the AUC of the combined model was higher than that of any single sequence radiomics model, and the difference was statistically significant. It indicated that the combined model based on multiple sequences improved diagnostic performance, and the inclusion of features from various new MRI techniques improved the model’s predictive ability.

The nomogram integrates the functional information from the CBF map, APTW map, QSM map, ADC map, and the patient’s IDH1 genotype to predict the individual probability of glioma recurrence. The results of this study showed that the nomogram had the largest AUC and high discrimination ability among all the models, with an AUC of 0.983 in the training cohort, 0.958 in the validation cohort and 0.972 in the temporal test. The lack of a significant difference in AUC between the nomogram and the combined radiomics model suggests that the predictive performance of the nomogram was driven predominantly by the radiomics component, whereas the incremental contribution of IDH1 was limited. The variability in the association of IDH1 across cohorts may partly account for its limited incremental benefit. The slightly higher AUCs observed in the temporal test cohort than in the validation cohort should be interpreted cautiously. This may reflect sampling variability related to the relatively small temporal cohort and potential differences in case mix between the two enrollment periods, rather than superior model performance or generalizability. DCA curves can show the net benefit of clinical decisions within a certain threshold probability, which can intuitively reflect the clinical value of the model (29). The DCA curve results of this study showed that the nomogram had the highest DCA curve in most of the threshold probability range, providing the greatest net benefit for identifying the recurrence. Therefore, the nomogram has the highest clinical value. In addition, the calibration curve of the nomogram indicated a good agreement between the predicted and actual probabilities of the nomogram. The Hosmer-Lemeshow test showed that the nomogram was a reliable and stable model with good fit. Using the nomogram presented in this study, it is possible to distinguish between recurrence and response to treatment in an efficient, immediate, and visible manner.

Several aspects of this study need to be improved. For a sample size of the training cohort, a minimum of 10 to 15 observations per predictor was required for logistic-regression analyses to produce reliable and stable estimates (30, 31). Firstly, for the final regression model, the training cohort of 147 patients provided more than 10–15 observations per retained predictor, given that six radiomics features were included in the combined model. However, this rule applies only to the final model-fitting stage and does not establish sample-size adequacy for the preceding high-dimensional feature discovery process, in which 4255 candidate features were initially evaluated. Although FDR correction, LASSO selection, and independent validation were used to reduce overfitting, some degree of feature-selection instability may remain. In addition, this was a retrospective single-center study, and all MRI examinations were performed on the same 3.0 T Philips platform. Although this ensured relatively homogeneous image acquisition, it may limit the generalizability of the model to other institutions, scanner vendors, and imaging protocols. Moreover, the temporal test cohort was enrolled during a later, non-overlapping period but was derived from the same institution and scanner platform; therefore, it represents temporal validation rather than true external validation. Larger multicenter cohorts are therefore needed to further validate the robustness of the selected radiomics signature.

Secondly, this study only focused on the tumor region. Glioma can easily infiltrate the surrounding normal brain tissue, and the peritumoral edema area can also reflect the heterogeneity of glioma (32, 33). In view of this, future research should include the peritumoral edema area as a focus area. In addition, MGMT promoter methylation status was not included in the present analysis. Given its established association with treatment-related imaging changes, particularly pseudoprogression after chemoradiotherapy, the absence of MGMT status may have introduced residual molecular confounding. Future studies incorporating MGMT promoter methylation and other relevant molecular markers are warranted to further assess their incremental value beyond the radiomics signature.

Thirdly, another limitation concerns the reference standard. Histopathological confirmation was not uniformly available and was more frequent in recurrence than in treatment-related response, reflecting routine clinical practice in which repeat surgery or biopsy is more commonly performed for suspected recurrence. In patients without pathological confirmation, longitudinal clinical-radiological assessment according to the RANO criteria was used, an approach widely adopted in glioma response assessment and previous MRI radiomics studies (28, 34, 35). Nevertheless, differential verification bias and some degree of incorporation bias cannot be completely excluded. Importantly, CBF, APTW, QSM, and ADC images were not used for the RANO-based reference-standard adjudication, and CE-T1WI features were not retained in the final combined model. Because the final model was based exclusively on CBF, APTW, QSM, and ADC radiomics features, the direct overlap between the imaging-based reference standard and the predictors included in the final model was substantially reduced, although not completely eliminated. Similar ground-truth challenges in postoperative glioblastoma surveillance have also been highlighted in recent probability-calibrated radiomics research (36). Future prospective multicenter studies using a more uniform and independent reference standard are warranted.

## Conclusions

Compared with the single sequence MRI radiomics model, the combined model has higher diagnostic efficiency. The information of ASL, APTW, QSM and DWI can complement each other, improving the model’s discrimination ability. The diagnostic efficacy of the nomogram was similar to that of the combined model, and it had a good differential effect in distinguishing recurrence from response to treatment. However, the nomogram may further facilitate individualized clinical assessment.

## Data Availability

The datasets generated and/or analyzed during the current study are not publicly available due to patient privacy and ethical restrictions, but anonymized data may be available from the corresponding author upon reasonable request and subject to approval by the relevant institution. Requests to access the datasets should be directed to lianghx@sd-cancer.com.
