# Supplementary material for: Multiparametric MRI radiomics integrating Arterial Spin Labeling, Quantitative Susceptibility Mapping, and Amide Proton Transfer imaging for differentiating glioma recurrence from post-treatment response
Source: Front Oncol. 2026 Sep 17;16:1956583. doi: 10.3389/fonc.2026.1956583 (PMC13627040; doi:10.3389/fonc.2026.1956583)
Supplement: Supplementary file 1 [file SupplementaryFile1.docx]

**Criteria of follow-up**

Reference-standard classification was based on histopathological findings or longitudinal imaging follow-up according to the Response Assessment in Neuro-Oncology (RANO) criteria^[1]^.

Patients were classified into the recurrence group if either of the following conditions was met: (1) histopathological examination after biopsy or second surgery confirmed viable glioma; or (2) longitudinal MRI follow-up, comprising at least two examinations over a follow-up period of ≥6 months, demonstrated progressive disease. Imaging evidence of progression included a ≥25% increase in the sum of the products of perpendicular diameters of measurable enhancing lesions compared with the smallest previous tumor measurement, the appearance of a new enhancing lesion, or a significant increase in T2-FLAIR/T2WI abnormality. For suspicious new or enlarging enhancement occurring within 12 weeks after completion of chemoradiotherapy, recurrence was not assigned on the initial MRI alone; longitudinal follow-up was continued, and only lesions showing persistent enlargement or subsequent unequivocal progression were classified as recurrence unless viable tumor was confirmed histopathologically.

Patients were classified into the post-treatment response group if either of the following conditions was met: (1) histopathological examination after biopsy or second surgery showed no viable glioma and findings consistent with treatment-related changes or necrosis; or (2) longitudinal MRI follow-up, comprising at least two examinations over a follow-up period of ≥6 months, demonstrated stable or decreased enhancing lesions on CE-T1WI and stable or decreased abnormalities on T2-FLAIR/T2WI, without subsequent imaging evidence of tumor progression.

For pathology-confirmed cases, the final histopathological diagnosis reported by the Department of Pathology was used as the reference standard. The pathologists were blinded to the corresponding MRI images, imaging-based classification, radiomics results, and model predictions, and were not involved in ROI segmentation, radiomics feature extraction, or model development.

1.	Wen PY, van den Bent M, Youssef G, Cloughesy TF, Ellingson BM, Weller M, et al. RANO 2.0: Update to the Response Assessment in Neuro-Oncology Criteria for High- and Low-Grade Gliomas in Adults. J Clin Oncol. 2023;41(33):5187-99.
