# Supplementary material for: Multiparametric MRI radiomics integrating Arterial Spin Labeling, Quantitative Susceptibility Mapping, and Amide Proton Transfer imaging for differentiating glioma recurrence from post-treatment response
Source: Front Oncol. 2026 Sep 17;16:1956583. doi: 10.3389/fonc.2026.1956583 (PMC13627040; doi:10.3389/fonc.2026.1956583)
Supplement: Supplementary file 2 [file SupplementaryFile2.docx]

MRI images were acquired with a Philips 3.0T MRI imaging system (Ingenia CX,Philips Healthcare,Best,the Netherlands). The head coil was a 32-channel coil. All patients underwent CE-T1WI, 3D-PcASL, 3D-APTW, 3D-GRE and DWI sequences.

The scanning parameters of CE-T1WI sequence were as follows: TR=600ms, TE=10ms, FOV=230mm×180mm, slice number =28, slice thickness =4mm, voxel size =0.9mm×1.1mm×4.0mm, Gd-DTPA was selected as the contrast agent, and the dose was 0.1mmol/kg. Contrast was injected through the cubital vein at a flow rate of approximately 3ml/s.

The 3D-pCASL sequence was used to reconstruct CBF maps. The scanning parameters were as follows: TR=4034ms, TE=10ms, FOV=240mm×240mm, slice thickness =6.0mm, slice number =14, matrix =64×60, flip Angle = 90°, excitation times 8, and post-labeling delay time 1.8s. The acquisition time was 4 minutes and 42 seconds, the scanning range covered the whole lesion, and the scanning direction was perpendicular to the common carotid artery. CBF maps can be displayed in pseudo-color on a Philips post-processing workstation (IntelliSpace Portal,version 7, Philips Healthcare,Best,the Netherlands). The order of red, yellow, green, and blue on the false-color image represents the change from high to low perfusion.

3D-APTW scanning parameters were as follows: TR=5925ms, TE=7.8ms, FOV=230mm×180mm, voxel size =1.8mm×1.8mm×6.0mm, slice number =14, slice thickness =6mm, matrix =252×220, sensitivity coding factor =1.6, acquisition time =5 minutes and 25 seconds. The scanning range covered all lesions. Seven 3D-FSE sequences with different frequency shifts (±3.1, ±3.5, ±3.9 and -1560 PPM) were used to reconstruct 3D-APTW images. Three image acquisitions at +3.5ppm were performed in order to calculate the B0 field map, which was used for B0 correction. The Radio Frequency (RF) saturation duration was 2.0s and the B1 power was 2.0μT. The application of a patient adaptive RF shim (MultiTransmit 4D) provides the same RF transmission when switching between the two channels and achieves optimal APTW imaging. APTW images can also be displayed in pseudo-color on a Philips post-processing workstation (IntelliSpace Portal,version 7, Philips Healthcare, Best, the Netherlands). On the pseudo-color image, the order of red, yellow, green, and blue represents the signal of the original image from high to low.

3D-GRE sequence was used to acquire phase and amplitude maps required for QSM image reconstruction. The scanning parameters were as follows: TR=43.0ms, TE of 10 groups were 6.69ms, 10.75ms, 14.81ms, 18.87ms, 22.93ms, 26.99ms, 31.05ms, 35.11ms, 39.17ms, 43.23ms, respectively. Flip Angle =15°, FOV=256mm×256mm, voxel size =1.0×mm×1.0mm×2.0mm, slice thickness =2.0mm, slice number =70, acquisition time = 4 minutes 32 seconds, anterior and posterior combined as the baseline scanning, scanning range of the whole brain. The original phase and amplitude maps were processed by MATLAB (Ver.2017b, Matrix Laboratory,Math Works) software and STI_Suite_V3.0 (Provided by Chunlei Liu) software to obtain QSM images.

The DWI sequence was used to reconstruct the ADC map, and the scanning parameters were as follows: DWI parameters: TR=2025ms, TE=71ms, b value 0,1000 s/mm², FOV=230mm×230mm, matrix =128×98, reconstruction matrix =256, slice number =20, slice thickness =5.5mm, acquisition time 12s.
