# Supplementary material for: Multiparametric MRI radiomics integrating Arterial Spin Labeling, Quantitative Susceptibility Mapping, and Amide Proton Transfer imaging for differentiating glioma recurrence from post-treatment response
Source: Front Oncol. 2026 Sep 17;16:1956583. doi: 10.3389/fonc.2026.1956583 (PMC13627040; doi:10.3389/fonc.2026.1956583)
Supplement: Supplementary file 3 [file SupplementaryFile3.docx]

**Supplementary Material 3. Completed CLEAR Checklist**

*Multiparametric MRI Radiomics Integrating Arterial Spin Labeling, Quantitative Susceptibility Mapping, and Amide Proton Transfer Imaging for Differentiating Glioma Recurrence from Post-treatment Response*

The checklist below was completed according to the CheckList for EvaluAtion of Radiomics research (CLEAR). “Yes” indicates that the requested information is reported in the manuscript or supplementary materials; “No” indicates that the item is not fully reported or publicly available; “N/A” indicates that the item is not applicable to the present study.

| **Section** | **No.** | **CLEAR reporting item** | **Yes** | **No** | **N/A** | **Page / location** |
| --- | --- | --- | --- | --- | --- | --- |
| **Title** | 1 | Radiomics methodology identified in the title | **Yes** |  |  | p. 1 |
| **Abstract** | 2 | Structured abstract summarizing study design, methods, results, and conclusions | **Yes** |  |  | pp. 1-2 |
| **Keywords** | 3 | Radiomics-relevant keywords provided | **Yes** |  |  | p. 2 |
| **Introduction** | 4 | Clinical/scientific background and knowledge gap described | **Yes** |  |  | pp. 4-5 |
|  | 5 | Rationale for using radiomics explained | **Yes** |  |  | pp. 4-5 |
|  | 6 | Study objectives stated | **Yes** |  |  | p. 5 |
| **Methods - Study design** | 7 | Use of a radiomics reporting guideline/checklist stated | **Yes** |  |  | Methods - Statistical Analysis, p. 12 |
|  | 8 | Ethics approval and consent information reported | **Yes** |  |  | p. 6; Declarations, p. 24 |
|  | 9 | Sample-size considerations described | **Yes** |  |  | Discussion - Limitations, pp. 20-22 |
|  | 10 | Retrospective study design identified | **Yes** |  |  | p. 6 |
|  | 11 | Inclusion/exclusion criteria and patient selection described | **Yes** |  |  | pp. 6-7; Fig. 1 |
|  | 12 | Technical radiomics workflow shown in a flowchart | **Yes** |  |  | Fig. 2; legend pp. 27 |
| **Methods - Data** | 13 | Institutional/private data source identified | **Yes** |  |  | Patients, p. 6 |
|  | 14 | Potential overlap with datasets used in previous publications addressed |  | **Yes** |  | Not stated |
|  | 15 | Training/validation/temporal split methodology described | **Yes** |  |  | p. 6; Results p. 12 |
|  | 16 | MRI acquisition and image-processing protocol reported | **Yes** |  |  | p. 8-10; Supplementary Material 2 |
|  | 17 | Non-radiomic predictors defined | **Yes** |  |  | p. 8 |
|  | 18 | Reference standard/outcome definition reported | **Yes** |  |  | pp. 7-8; Supplementary Materials 1 and 4 |
| **Methods - Segmentation** | 19 | Segmentation strategy and target region described | **Yes** |  |  | pp. 8-9 |
|  | 20 | Segmentation operators and experience described | **Yes** |  |  | p. 9 |
| **Methods - Pre-processing** | 21 | Image pre-processing steps reported | **Yes** |  |  | pp. 8-10 |
|  | 22 | Resampling approach and voxel size reported | **Yes** |  |  | pp. 8-10 |
|  | 23 | Gray-level discretization method and bin width reported | **Yes** |  |  | p. 10 |
|  | 24 | Image types/transforms used for radiomics described | **Yes** |  |  | p. 10; Table 3, p. 31 |
| **Methods - Feature extraction** | 25 | Feature-extraction software, versions, and radiomics approach reported | **Yes** |  |  | pp. 9-10 |
|  | 26 | Radiomic feature classes and counts reported | **Yes** |  |  | p. 10 |
|  | 27 | Total number of extracted features per sequence and overall reported | **Yes** |  |  | p. 10 |
|  | 28 | Statement that all unspecified extraction parameters remained at software defaults |  | **Yes** |  | Not stated |
| **Methods - Data preparation** | 29 | Presence/absence and handling of missing data reported |  | **Yes** |  | Not stated |
|  | 30 | Class imbalance and any balancing strategy explicitly addressed |  | **Yes** |  | Class counts reported on pp. 12-13; balancing strategy not stated |
|  | 31 | Intra-/inter-observer segmentation reliability procedure reported | **Yes** |  |  | p. 9 |
|  | 32 | Feature scaling/standardization and training-only parameter estimation reported | **Yes** |  |  | p. 10 |
|  | 33 | Dimensionality-reduction/feature-selection procedure reported | **Yes** |  |  | pp. 10-11 |
| **Methods - Modeling** | 34 | Modeling algorithms and software/packages described | **Yes** |  |  | pp. 11-12 |
|  | 35 | Training/tuning procedure, including LASSO cross-validation and lambda.min, reported | **Yes** |  |  | pp. 10-11 |
|  | 36 | Potential confounding clinical variables considered/adjusted and limitations discussed | **Yes** |  |  | p. 8; Discussion pp. 17-18 and 20-22 |
|  | 37 | Model/feature selection strategy described | **Yes** |  |  | pp. 10-11, 14-16 |
| **Methods - Evaluation** | 38 | Internal and temporal testing strategy described | **Yes** |  |  | p. 6; Results, p. 12 |
|  | 39 | Performance metrics and explicit rationale for metric selection reported |  | **Yes** |  | Metrics reported on pp. 11-12; rationale not explicitly stated |
|  | 40 | Uncertainty measures (95% confidence intervals) reported | **Yes** |  |  | Table 4, pp. 32-34 |
|  | 41 | Statistical comparison of model performance reported | **Yes** |  |  | pp. 11, 15-16; Table 5, p. 35 |
|  | 42 | Radiomics models compared with clinical and combined approaches | **Yes** |  |  | pp. 14-16; Tables 4-5, pp. 32-35 |
|  | 43 | Model interpretability provided through explicit Rad-score formula and nomogram | **Yes** |  |  | p. 16; Fig. 6 |
| **Results** | 44 | Baseline demographic and clinical characteristics reported by cohort/group | **Yes** |  |  | pp. 13-14; Table 1, p. 29 |
|  | 45 | Patient eligibility/recruitment flowchart provided | **Yes** |  |  | Fig. 1 |
|  | 46 | Feature reproducibility/selection statistics and final feature set reported | **Yes** |  |  | pp. 14-15; Table 3, p. 31 |
|  | 47 | Model performance reported for training, validation, and temporal test cohorts | **Yes** |  |  | pp. 15-16; Tables 4-5; Figs. 5-8 |
|  | 48 | Clinical, single-sequence, combined, and nomogram approaches compared | **Yes** |  |  | pp. 14-16; Tables 4-5 |
| **Discussion** | 49 | Main findings summarized and interpreted | **Yes** |  |  | pp. 17-20 |
|  | 50 | Findings compared with relevant previous radiomics studies | **Yes** |  |  | pp. 18-19 |
|  | 51 | Clinical/practical implications discussed | **Yes** |  |  | pp. 17-20; Conclusions p. 23 |
|  | 52 | Bias, overfitting, generalizability, reference-standard, and molecular limitations discussed | **Yes** |  |  | pp. 20-22 |
| **Open Science - Data** | 53 | Raw/processed images and segmentation data publicly shared |  | **Yes** |  | Data availability statement, p. 24; not publicly deposited |
|  | 54 | Radiomic feature dataset publicly shared |  | **Yes** |  | Data availability statement, p. 24; available on reasonable request, not publicly deposited |
| **Open Science - Code** | 55 | Pre-processing and feature-extraction settings/scripts shared or fully described | **Yes** |  |  | pp. 9-11; Supplementary Material 2 |
|  | 56 | Modeling source code publicly shared |  | **Yes** |  | No public code repository reported |
| **Open Science - Model** | 57 | Final trained model file publicly shared |  | **Yes** |  | Rad-score formula reported on p. 16; model file not shared |
|  | 58 | Ready-to-use software/tool made available |  |  | **N/A** | No standalone system was developed |

**Reference:** Kocak B, Baessler B, Bakas S, et al. CheckList for EvaluAtion of Radiomics research (CLEAR): a step-by-step reporting guideline for authors and reviewers endorsed by ESR and EuSoMII. Insights Imaging. 2023;14(1):75. doi:10.1186/s13244-023-01415-8.
