# Supplementary material for: Multiparametric MRI radiomics integrating Arterial Spin Labeling, Quantitative Susceptibility Mapping, and Amide Proton Transfer imaging for differentiating glioma recurrence from post-treatment response
Source: Front Oncol. 2026 Sep 17;16:1956583. doi: 10.3389/fonc.2026.1956583 (PMC13627040; doi:10.3389/fonc.2026.1956583)
Supplement: Supplementary file 4 [file SupplementaryFile4.docx]

**The ratio of reference-standard verification**

| **Cohort** | **Diagnostic group** | **Histopathology, n (%)** | **RANO criteria, n (%)** | **Total** |
| --- | --- | --- | --- | --- |
| Training | Recurrence | 36 (37.1%) | 61 (62.9%) | 97 |
| Training | Treatment response | 6 (12.0%) | 44 (88.0%) | 50 |
| Validation | Recurrence | 15 (36.6%) | 26 (63.4%) | 41 |
| Validation | Treatment response | 2 (9.1%) | 20 (90.9%) | 22 |
| Temporal test | Recurrence | 16 (36.4%) | 28 (63.6%) | 44 |
| Temporal test | Treatment response | 2 (10.0%) | 18 (90.0%) | 20 |
| Total | Recurrence | 67 (36.8%) | 115 (63.2%) | 182 |
| Total | Treatment response | 10 (10.9%) | 82 (89.1%) | 92 |

**Overall pathology verification rate by cohort**

| **Cohort** | **Pathology** | **Total** | **Pathology verification rate** |
| --- | --- | --- | --- |
| Training | 42 | 147 | 28.6% |
| Validation | 17 | 63 | 27.0% |
| Temporal | 18 | 64 | 28.1% |

The overall pathology verification rates were 28.6%, 27.0%, and 28.1% in the training, validation, and temporal test cohorts, respectively.
